# The information overload, immunology and their relationship to world health

**DOI:** 10.1098/rsob.240392

**Published:** 2025-05-07

**Authors:** Peter A. Bretscher

**Affiliations:** ^1^ Department of Biochemistry, Microbiology and Immunology, University of Saskatchewan, Saskatoon, Saskatchewan, Canada

**Keywords:** information overload, world health, tuberculosis, AIDS, cancer, vaccination

## Abstract

Essential to much medical progress (for example, preventing AIDS, tuberculosis and cancer) is understanding how the class of immunity is controlled. Most envisage that pathogen- or danger-associated signals are critical. Many classical observations, particularly on the variables of immunization affecting the class of immunity generated, are paradoxical under this dominant view, and are therefore neglected. Among these is the role of antigen dose. Plausible strategies to prevent and treat AIDS, cancer and tuberculosis are based on such neglected observations. Many suggest that the information overload stultifies the canon, inhibiting progress. I illustrate here that seeking and resolving paradoxes can provide a different perspective from that of the dominant canon, opening vistas that address major issues pertinent to world health.

**Figure:**
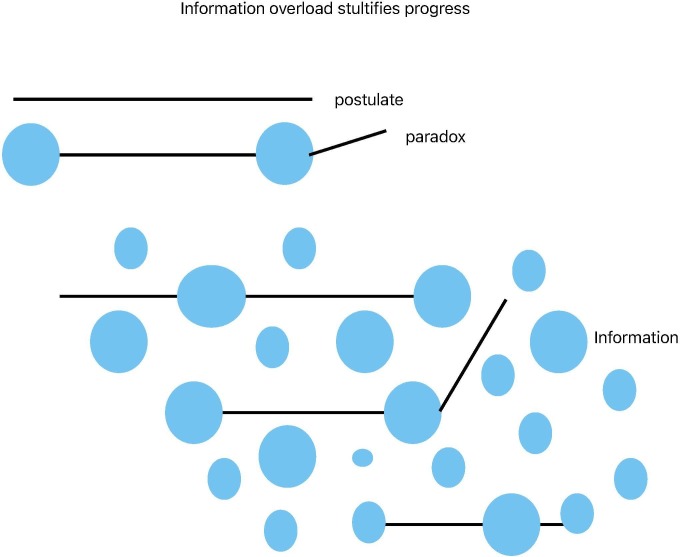

## 1. Prologue

E. O. Wilson, the biologist, said ‘We are drowning in information, while starving for wisdom’. The existence over decades of a considerable number of observations in the literature—paradoxical in the context of the frameworks dominating immunological research and therefore neglected—attests to this reality [1,2]. Not all is well in the state of Denmark. These frameworks have provided the context for attempts to develop strategies of immunological intervention to address major problems of world health. The lack of success of this agenda is apparent. The nature of the needs to prevent and treat several major diseases is evident, but strategies to meet these needs have not been realized over decades. I argue here for both the gravity of the situation and for ways of transcending it.

This article is illustrative rather than comprehensive. I focus on one foundational question. I address the significance of the answer to this question in the context of developing strategies against AIDS, cancer and tuberculosis. Such a focus avoids the complexities that arise from a comprehensive description of the immune system.

The focused question I consider here is what determines whether foreign antigens generate a predominant and stable cell-mediated Th1 immune response, or one that in time evolves to contain a substantial or predominant antibody Th2 component [3]. Clinical and experimental observations attest to the centrality of this question. I avoid, to maintain a focus, discussion of how other classes/subclasses of immunity are differentially regulated. I have outlined my broader perspective elsewhere [4].

## 2. The physiological and clinical importance of immune class regulation

It was first appreciated in the 1960s that most experimental tumours are contained by cell-mediated immunity; tumour progression often correlated with antibody production and the associated downregulation of the cell-mediated response [5]. Modern findings support the generalization that a sufficiently strong cell-mediated response contains most cancers [6]. Cancer progression is associated with a cell-mediated response that is too weak. This is probably due either to the poor immunogenicity of the cancer or to the downregulation of protective cell-mediated immunity as the anti-cancer response evolves into a different mode.

Those individuals (about 1%) who, upon infection with HIV-1, generate a stable Th1 cell-mediated response (the elite controllers) do not develop AIDS [7]. The majority, who if untreated develop AIDS, do not suffer serious symptoms shortly after infection, when an exclusive cell-mediated response is evident. They develop pathological symptoms once they produce antibodies, associated with a mixed Th1/Th2 or predominant Th2 response [8]. Two conclusions seem plausible. An exclusive cell-mediated response contains HIV-1. All infected individuals have the genetic capacity to generate such a response, as they do so shortly after infection.

An explanation for these immune correlates of protection following HIV-1 infection is generally accepted. HIV-1 has mechanisms for rapidly producing viral variants. Antibodies produced against the viral strain responsible for the initial infection, and able to neutralize this strain, are often unable to neutralize some of the variants generated. These ‘escape variants’ undermine the efficacy of the antibody response. This interpretation is supported by the finding that some HIV-1-infected individuals, who control the viral load better than most, produce antibodies capable of neutralizing diverse viral variants [9].

Tuberculosis is still among the most devastating infectious diseases worldwide. Despite this, more than 90% of those infected by *Mycobacterium tuberculosis* resist the infection and remain healthy [10]. Many observations indicate that cell-mediated immunity is protective, whereas other observations appear paradoxical under this view [11]. We recently argued that these paradoxes are accounted for by the hypothesis of there being two types of tuberculosis [12]. In type I, the pathogen is insufficiently immunogenic to generate the cell-mediated response required to contain the infection. In type II, the cell-mediated response is too weak due to its downregulation as the immune response evolves towards a Th2 mode.

These observations on immunity against cancers, HIV-1 and *M. tuberculosis* make clear how prevention of disease may be achieved. Guaranteeing a strong and stable cell-mediated Th1 response on natural infection, or when a cancer arises, should prove efficacious. Efficacious treatment of disease would depend on the nature of the failure of the immunity, generated upon infection, to contain the pathogen or cancer. The patient’s cell-mediated immunity may be inadequate if the cancer or pathogen is insufficiently immunogenic. Cure would be achieved by increasing the intensity of the cell-mediated response. Alternatively, the patient’s cell-mediated immunity may be inadequate due to the response being in a different mode, associated with the downregulation of cell-mediated immunity. In this case, regulating the immune response so it resolves into a predominant cell-mediated mode would probably constitute treatment. The critical importance of knowing the variables of immunization that affect the cell-mediated/antibody nature of the ensuing response, to achieve a strong cell-mediated response, is clear.

## 3. Variables of immunization affecting the Th1/Th2 phenotype of the ensuing response

Two variables have been evident for over six decades. Moreover, the centrality of these variables in affecting the nature of the response against diverse types of antigens has become ever more compelling. The first variable is time after antigen impact [13]. As already indicated, an exclusive cell-mediated response is most often first generated, and this response often evolves over time towards a Th2, antibody mode, associated with the downregulation of cell-mediated immunity. This pattern is observed in the response to proteins [12], more complex antigens such as sheep erythrocytes in mice [14], and in responses to viruses [7], bacteria [15], protozoa [16,17] and tumours [18]. The second variable is the amount of antigen, for non-replicating antigens, or the number of replicating entities, for slowly replicating antigens. Low amounts or numbers generate an exclusive cell-mediated response. Medium amounts and numbers generate an exclusive cell-mediated immune response more rapidly; this response evolves with time towards an antibody, Th2 mode. Even higher amounts or numbers generate more rapid responses, and the cell-mediated phase may be transient or altogether eclipsed. This same pattern is seen in responses to diverse antigens: proteins [13], sheep erythrocytes in mice [14], viruses [7], protozoa [16,17], bacteria [15] and tumours [17]. Does this pattern also hold for different routes of immunization? Much evidence supports an affirmative answer. For example, we examined whether this pattern held in mice for the antigen BCG, employed to vaccinate people against tuberculosis, when immunization was by the intravenous, subcutaneous and intradermal routes. The observations obtained again attest to the generality of the dependence of the nature of the immune response on antigen dose [15].

I outline in this article the frameworks employed by most in addressing the primary question outlined above. I do not attempt to address why most think this framework plausible, but I do indicate some grounds for why I find it wanting.

## 4. Frameworks for understanding how the Th1/Th2 phenotype is determined

Janeway [19,20] and Matzinger [21–23] have argued that the immune system does not discriminate self from non-self (foreign) antigens but, rather, in the case of Janeway, the immune system responds only to, or in the presence of, material derived from infectious entities; in the case of Matzinger, it responds to antigen only when the antigen impacts the immune system under ‘dangerous’ circumstances. An encapsulation of their conclusions is that the activation of CD4 T cells, and thus the initiation of all immune responses, requires a *pattern recognition receptor* (*PRR)* to bind to a *pathogen-associated molecular pattern* (*PAMP*) (Janeway), or to a *danger-associated molecular pattern* (*DAMP)* (Matzinger). In the absence of a PAMP- or DAMP-initiated signal, antigen can inactivate the CD4 T cells [24]. In addition, most argue that the particular nature of the PAMP/DAMP signals also determines the Th subset to which the activated Th cells belong, e.g. whether Th1 or Th2 cells are primarily generated [3,25–28]. I refer to this framework as the DAMP/PAMP-centric view.

## 5. Paradoxes within the context of the DAMP/PAMP view

Many classical findings are paradoxical in the context of this view. Firstly, the PAMP signals do not change with time after antigen impact, yet the Th1/Th2 nature of the response to diverse antigens does. Secondly, the dependency of the Th1/Th2 phenotype of the ensuing response on the amount of antigen is true for diverse antigens: foreign vertebrate antigens expected to be PAMP-free, and tumours, viruses, bacteria and protozoa that are expected to express very different PAMPs. A plausible explanation for this dependence should presumably be PAMP-independent. Moreover, these different amounts of antigen, within one experimental setting, are injected under identical circumstances, presumably provoking identical ‘danger’ signals. The different natures of the ensuing immune responses are again paradoxical in the context of the danger model and problematic for the PAMP model [29]. I point out further paradoxes below. These paradoxes have long existed and yet seem to have caused most researchers little concern.

I should stress, though, that the view expressed here does not mean that foreign vertebrate antigens cannot contain components that activate receptors involved in innate immunity and thereby affect the adaptive response. For example, it appears that RNA expressed inside intact sheep red blood cells (SRBC) can activate innate pathways of those APCs that engulf these SRBC, generating stronger antibody responses against the antigen [30]. Despite this, the Th1/Th2 phenotype of the response to SRBC follows the general pattern outlined above in its dependence on the dose of antigen and time after immunization [14,15]. Thus, the Th1/Th2 phenotype of the response still appears to be controlled by the threshold mechanism, described immediately beliow.

## 6. The threshold hypothesis

I proposed a hypothesis in 1974—the threshold hypothesis—to explain how the Th1/Th2 phenotype of the immune response is determined [31]. This hypothesis was quantitative and accounted for all the quantitative variables of immunization then known, and as outlined above, that affect the Th1/Th2 phenotype of the ensuing response. It was natural to suppose that these variables, accounted for by the hypothesis, also governed the nature of the response to diverse antigens, not yet examined, as indeed further observations have amply demonstrated. I do not outline the rationale for, or the details of, this hypothesis here. However, the hypothesis makes one striking prediction that I feel I should state and whose consequences I should discuss. Consider a situation where the administration of antigen results in an immune response with a substantial Th2 component. The prediction is that partial depletion of CD4 T cells, around the time of antigen impact, will deviate the immune response generated towards a Th1 mode. This prediction has been successfully tested in diverse systems [32]. It presents an acute paradox for the PAMP/DAMP-centric view. Depletion of CD4 T cells is not anticipated to change the DAMP or PAMP signals; thus, the modulation of the immune response observed is inexplicable on this view.

## 7. The generality of the Th1/Th2 phenotype of the immune response on the amount of antigen delivered

One value of a theory is that it provides grounds for believing in the generality of a particular observation that can be accounted for. The mouse model of cutaneous leishmaniasis is the best-studied animal model of an infectious disease uniquely contained by an exclusive/predominant cell-mediated, Th1 response. Mice of different strains, infected with a million of the pathogenic parasites, mount different types of response. Most generate a stable Th1 response, contain the parasite at low levels, and are therefore deemed resistant. In contrast, mice of the BALB/c strain rapidly generate a Th2 response, suffer uncontrolled parasitemia and are naturally deemed susceptible. The diversity of responses among mice of different strains to the standard challenge is striking [33]. We exploited this system to test the generality of the dependency of the Th1/Th2 phenotype of the response on the number of parasites employed in the infection. We found in all strains of mice that infection with relatively low and high numbers resulted, respectively, in a stable Th1 response and a response that, with time, developed a substantial/predominant Th2 component. We could define, for any mouse strain, a transition number, *N*_t_. Infection with a number below *N*_t_ results in a stable Th1, cell-mediated response; infection with a number above *N*_t_ results in a response that, in time, develops a substantial Th2 component. We were struck that the value of *N*_t_ varied, among mice of different strains, over a 100 000-fold range [17]. We realized this range reflected genetic diversity among the mouse strains, and that such diversity probably protects us, as a species, from novel pathogens. Such diversity results in diverse responses in different individuals, with a greater chance that at least some will survive. Note that only about 1% of those infected by HIV-1 are elite controllers. Our experimental studies on human tuberculosis provided us with indirect evidence that the value of *N*_t_ for *M. tuberculosis* infection of people also varies greatly [12]. This analysis, showing the great variability in *N*_t_ in some instances, has greatly affected our thinking.

## 8. Cell-mediated immune deviation

Studies by Parish in the late 1960s have also greatly influenced our thinking and experimental studies [34,35]. Parish showed that repetitive immunization with low amounts of a purified protein antigen, over some weeks, can result in a stable state of cell-mediated immunity. The immunized animals were challenged with an amount of antigen that generates an exclusive and brisk antibody response in a naïve animal; however, the immunized animals continued to express cell-mediated immunity and produced little antibody upon this challenge. Parish had apparently found a means of locking the immune response into a cell-mediated mode. We refer to this state as a state of cell-mediated immune deviation. My students and I explored whether similar states of cell-mediated immune deviation can be established by similar means against much more ‘complicated antigens’ than the purified protein employed by Parish. We have established such states in mice against the pathogen that causes cutaneous leishmaniasis in humans [16], against mycobacteria [36] and against experimental tumours [18]. These protocols would appear to provide models for creating effective vaccines against pathogens and cancers preferentially susceptible to cell-mediated attack. We refer to this protocol as the low-dose vaccination strategy. Buddle tested this strategy in cattle against tuberculosis, with outstanding success [37].

## 9. The possibility of strategies of universally efficacious vaccination

A major practical issue is how a standard vaccination strategy can be developed in the face of the genetic diversity of the vaccinated population. This diversity will be reflected in the variability of *N*_t_. I have suggested that an ultra-low-dose strategy can overcome this problem. Bacille Calmette Guerin (BCG) is an attenuated *Mycobacterium* employed to vaccinate against tuberculosis. A very large trial has shown that, as employed in the vaccination of people, BCG provides minimal, if any, protection. Buddle demonstrated that BCG vaccination could be highly efficacious in protecting cattle against an experimental challenge by the cattle pathogen when he employed a dose of BCG about a million-fold lower than that commonly used [37]. My strategy for a universally effective vaccination protocol relies on the fact that BCG replicates. I suggest that infection with a very low number of BCG, below the transition number of all individuals vaccinated, will initially not induce an immune response in some, and therefore the BCG will grow in an unrestrained manner. When the BCG reaches a level that induces a cell-mediated response, they will also generate a cell-mediated imprint [4]. Thus, infection with a very low number will generate a Th1 imprint in all.

## 10. The impact of the low-dose vaccination strategy

The promise that our low-dose vaccination strategy holds for preventing AIDS, tuberculosis and some cancers made me hopeful. It has been over three decades since we reported in *Science* [16] the first successful testing of this strategy in the mouse model of cutaneous leishmaniasis. Although this report has gained significant citations, its impact has been less than I had hoped for and anticipated. Reviews on the immunology of human tuberculosis have rarely, if ever, cited Buddle’s work, outlined above. The lack of influence of Buddle and of our work has caused me to reflect on why this might be so. At the same time, there has been increasing discussion in the literature regarding information overload and its impact in undermining research resilience [38–40]. I cannot help but think three significant generalizations are related. The first is what has been called ‘the ossification of the canon’ [38], or the difficulty, in the context of the information overload, in recognising and profiting from valuable ‘disruptive research’ [40]. This neglect, I suggest, is bound to result in the accumulation of paradoxes. I suggest, indeed, that seeking out paradoxes and attending to their resolution is a realistic way of transcending the information overload [9]. These two generalizations may well be related to the third, which addresses the primary subject of this article. I have outlined above how many observations regarding the nature of the variables of immunization that affect the Th1/Th2 nature of the response are paradoxical in the context of the dominant DAMP/PAMP perspective. These observations relate to the impact of antigen dose and time after antigen impact on the nature of the ensuing response. I suggest that observations paradoxical in the context of the dominant conceptual frameworks tend to be ignored and put aside by most researchers as not being of general significance. I have discussed with many older and younger colleagues whether they know of Parish’s studies, described above. No one I have asked has responded positively, except for one older colleague who could not quite clearly recall their nature or their potential import. It could be that Parish’s reported observations reflect *particular* circumstances of the animals and antigens employed. The collective we pay attention to observations only when we regard them as reflecting *generalities*. I think the following three statements, imagined by me, illustrate the mentality governing much contemporary immunological research. The dominant DAMP/PAMP framework is highly plausible. A report shows that low doses of sheep erythrocytes generate exclusive cell-mediated responses, and higher doses induce antibodies. These observations are not readily explicable in the DAMP/PAMP framework. They are therefore probably idiosyncratic findings, particular to the experimental system employed. The grant proposal to try to establish a cell-mediated imprint based on Parish’s findings is not worthy of support. The general significance and pertinence of Parish’s findings, made over 50 years ago, are highly questionable, as they have not been repeated in other experimental systems. This last statement is not true, of course. It is an attempt to capture a typical thought of a contemporary researcher.

## 11. The potential importance of Parish’s study

I sometimes wonder what would have occurred if Parish’s findings had been taken more seriously by more people. We did, and our small group found we could generate low-zone cell-mediated imprints against protozoa, bacteria and cancers, as described above. I still think the ultra-low-dose strategy will, in time, contribute to vaccination against entities preferentially susceptible to cell-mediated attack.

No framework is complete, and so, in an absolute sense, none is correct. However, some frameworks are clearly wrong and, if widely and dominantly held, present a barrier to progress. I regard the DAMP/PAMP perspective as such a framework. A more valid framework tends to open up new avenues of investigation and insight. I would like to round off this article by indicating how The Threshold Hypothesis has stimulated me and my colleagues to theoretically explore and experimentally test strategies to treat AIDS, tuberculosis and cancers.

## 12. Treatment of disease

It is generally accepted that the pathogen causing human visceral leishmaniasis, a deadly disease unless treated, can be contained by a predominant cell-mediated response against the pathogen. The healthy infected express strong cell-mediated immunity and produce low levels of antibody. Patients with disease, in contrast, express little cell-mediated immunity and produce more antibody. This disease is rapidly fatal if untreated. Standard treatment consists of the administration of an anti-parasitic drug for a few weeks. Treatment results in a re-expression of cell-mediated immunity and lower levels of antibody [41]. We showed that the IgG antibody of the healthy infected and drug-cured individuals shared characteristics associated with a predominant Th1 response, in contrast to the antibody of patients before treatment, as reflected in the IgG subclass to which the antibody predominantly belonged [41]. Most interestingly, treated patients are known to be resistant to developing disease again, even though living in an endemic area. The immunity of treated individuals was indistinguishable from that of the healthy infected [41]. The drug treatment killed the parasite and so reduced the parasite load. We have argued, on the basis of these and other observations [42], that antigen load is not only critical in determining the Th1/Th2 phenotype of primary immune responses but can also control the Th1/Th2 phenotype of ongoing immune responses.

I would like to take a break from science here. I find the situation remarkable. The evidence seems overwhelming that individuals infected by HIV, who make a stable and predominant Th1 response, the elite controllers, contain the infection, whereas the response of most infected and untreated individuals develops a substantial antibody Th2 component, leading to AIDS. There seems little, if any, discussion in the literature on whether the approach standardly employed to treat visceral leishmaniasis might have lessons for controlling HIV-1 replication in AIDS patients. This neglect might not be so prevalent if visceral leishmaniasis was more prevalent in the ‘more civilized’ parts of the world! This treatment against visceral leishmaniasis is, I believe, the first against any infectious disease caused by a pathogen preferentially susceptible to cell-mediated attack.

## 13. Treatment of HIV infections

Moreover, as anti-retroviral treatment reduces viral load, I expect such treatment, administered to an HIV-1 infected individual whose response has a significant Th2 component, to initially modulate the nature of the immune response towards the type of immunity evident in elite controllers [43]. Indeed, studies show that some individuals who have been on drug treatment for a considerable time, several years, do control the virus when treatment is stopped. This control is due, presumably, to an effective immune response. These individuals are referred to as post-treatment controllers [44]. While elite controllers constitute about 1% of HIV-1 infected individuals, post-treatment controllers constituted about 15% of the individuals involved in one study [44]. These observations appear to be generally regarded as enigmatic. We have suggested that they can be explained by the effect of anti-viral therapy on reducing antigen levels and the effect this has on the nature of the infected individual’s anti-viral immune response. Moreover, we have discussed how this hypothesis can lead to a simple and personalized form of treatment. Anti-retroviral therapy can be stopped at a time when we know the individual’s immune response is in an optimally protective, cell-mediated mode, expected to contain the virus. This state can be ascertained by the predominant presence of the subclass of IgG antibody characteristic of predominant Th1 responses. The treated individuals are likely to no longer be able to infect others [43].

## 14. Treatment of tuberculosis and cancers

Similar principles can govern the treatment of tuberculosis [45] and cancer [46]. All these approaches, outlined here to prevent and treat cancer, AIDS and tuberculosis, are built on what is now a large body of observations that show how important antigen dose is in controlling the nature of the immune response. Such observations are at least inexplicable, if not paradoxical, in the context of the dominant DAMP/PAMP perspective. They therefore tend to be ignored. I think this situation calls for a radical reassessment.

## Data Availability

This article has no additional data.
